# Effect of Oil Type Used in Neapolitan Pizza TSG Topping on Its Physical, Chemical, and Sensory Properties

**DOI:** 10.3390/foods12010041

**Published:** 2022-12-22

**Authors:** Amalia Piscopo, Angela Zappia, Antonio Mincione, Roccangelo Silletti, Carmine Summo, Antonella Pasqualone

**Affiliations:** 1 Department AGRARIA, University Mediterranea of Reggio Calabria, via dell’Università 25, 89124 Reggio Calabria, Italy; 2 Department of Soil, Plant and Food Science (DISSPA), University of Bari Aldo Moro, via Amendola, 165/a, 70126 Bari, Italy

**Keywords:** olive oil Ottobratica cv., oxidation, pizza, polar compounds, volatile compounds

## Abstract

Background: According to the regulations of the Neapolitan Pizza TSG, extra virgin olive oil must be exclusively used as topping ingredient, together with tomato for pizza marinara-type production. As, often deliberately, other oils are replaced by pizza makers for economical and organoleptic purposes, the present study was conducted to analyze the quality of pizza depending on the oil typology used. Methods: Chemical and sensory analyses were performed on olive oils and on pizza topping mix samples after cooking to detect changes due to the applied cooking processing. Results: The results revealed the best quality of a monovarietal olive oil (Ottobratica cv.) for their peculiar phenolic content related to the best oxidation stability after pizza’s cooking, expressed as bioactive amounts and lower presence of undesired volatile compounds. Conclusions: The use of an extra virgin monovarietal olive oil, such as Ottobratica cv., in the topping of pizza is preferable to other oils, also EVOO, because of its higher quality, which is reflected in greater health and pleasant characteristics from a sensorial point of view.

## 1. Introduction

Pizza is one of the most appreciated and popular Italian foods in the world. It is a salty gastronomic product consisting of a dough made from flour, water and yeast, flattened and stuffed with tomato, mozzarella and other ingredients. The origins are not clear. From the information available, the existence of a profession called *pizzaiuolo* in Naples in the south of Italy is certain, as attested in a document of 1799 [1]. Acquired nowadays by the modern pizza maker, the art of the Neapolitan pizzaiolo has been recognized as an intangible cultural heritage by UNESCO for the different stages of an ancient technique handed down for generations, which makes the dough light, highly digestible and tasty after cooking: the dough production, the modeling of the loaves before leavening, the creation of the pizza disk, its garnish and, finally, the control of oven cooking. This rigorous preparation falls into the preparation called “Neapolitan pizza,” recognized as a “Traditional speciality guaranteed” (TSG) since 5 February 2010, thanks to EC Regulation 97/2010 [2], which includes several specifications of productions. Two types of pizza are covered in this specification, depending on the topping ingredients: pizza marinara (garnished principally with tomato and extra virgin olive oil) and pizza margherita (with the addition of Mozzarella di Bufala Campana PDO or Mozzarella Fiordilatte TSG to the previous ingredients).

If on the one hand the ingredients of the pizza topping recall limits strongly linked to tradition, on the other hand nowadays, there is a risk of a general decrease in the quality of food production, including pizza, linked to the choice of adopting different and lower-cost ingredients in a market global in which “Made in Italy” foods are still highly successful and appreciated. Among the topping ingredients of pizza, particular attention was paid in this study to olive oil, which according to the production regulations of the “Neapolitan Pizza TSG,” must be exclusively of extra virgin category, but which is often deliberately replaced by pizza makers with other oils (such as seed ones) for economic and also organoleptic purposes. In fact, pizza consumption trends led to an increasing demand for different, elaborate and even sophisticated toppings (characteristics, for example, of the so-called gourmet pizzas) where the ingredients must be in balance in order to enhance some desired flavors.

The large oil production in Italy, in particular in the southern regions, offers a possibility of using olive oils that in their various peculiarities can give food preparations different sensory and nutritional characteristics. Lipids are an integral part of the human diet for their nutritional value, but also for their physiological and sensory properties [3,4]. Heating of oil produces various chemical changes, including oxidation: its fast rate involves unstable primary oxidation products, hydroperoxides, which are decomposed rapidly into secondary oxidation products, such as aldehydes and ketones. Moreover, volatiles such as aldehydes, ketones, short-chain hydrocarbons, lactones, alcohols, and esters are produced from hydroperoxide decomposition and many nonvolatile polar compounds and triacylglycerol dimers and polymers are produced in thermally oxidized oil by radical reactions [5]. Among oils, olive oil is more resistant to oxidation due to the greater presence of bioactive compounds such as polyphenols [6]. The absolute concentration of phenolic bioactive compounds in olive oil is the result of complex interactions among several factors, including cultivars, degree of ripeness, climate, and extraction process [7,8,9]. The oil obtained from the Ottobratica olive cultivar has been the subject of research for its qualitative characteristics [10,11], and in the present study its application has been evaluated as a distinctive ingredient for Neapolitan pizza in comparison with two other olive oils: a commercial extra virgin and a rectified one. To date, and to our knowledge, there are still no scientific works concerning studies on the use of olive oils of different quality to season pizza, so the aim of this work was to evaluate some effects on the overall (chemical and sensorial) characteristics of Neapolitan pizza TSG.

## 2. Materials and Methods

### 2.1. Sampling

The pizza samples were produced in a local pizzeria in Reggio Calabria according to the methods and with the raw materials described in the Production Regulations of the Pizza Napoletana TSG [2]. After the preliminary stages of preparation of the pizza and the drafting of the dough, all the pizza samples were seasoned with a tomato sauce obtained from San Marzano DOP tomatoes (salted), to which three types of olive oil were added: A (oil derived from refined and virgin olive oils), B (commercial extra virgin olive oil), and C (monovarietal extra virgin olive oil from Ottobratica cv.). After cooking, the pizza samples were transferred in appropriate paper packaging to the Food technology laboratory of the University “Mediterranea” of Reggio Calabria for the pizza topping analyses. From each pizza sample, cooked topping was taken and stored at −18 °C. Qualitative analyses were conducted on the single topping ingredients (tomato sauce and oils A, B, and C) and on the topping samples (tomato sauce + oil A/oil B/oil C) before and after oven cooking.

### 2.2. Topping Ingredient Analyses

The single topping ingredients (tomato sauce and oils A, B and C) were analyzed for their principal qualitative characteristics. For the olive oil samples, free acidity, peroxide number, and spectrophotometric indices were assessed following the European regulation [12,13]. The method reported by Baiano et al. [14] was followed for total phenol content quantification, and for the qualitative determination chromatographic analysis was performed. For this purpose, a UHPLC (Knauer) system with a Knauer column C18A (100 mm × 2 mm) was used, and the chromatographic separation occurred through a gradient of elution of two mobile phases: water acidified with acetic acid up to pH 3.1 and acetonitrile. The elution flow was maintained at 0.4 mL/min at a controlled temperature of 30 °C; 5 μL of sample, suitably filtered with 0.45 μm syringe filters were submitted for analysis. Calibration lines have been constructed for the identification of chromatographic peaks, obtaining a squared correlation factor of 0.999. The results were expressed in mg/kg.

The volatile compounds of olive oil samples were determined by headspace solid-phase microextraction (HS-SPME) coupled with gas chromatography–mass spectrometry (GC–MS). Samples were weighed (500 ± 0.05 mg) in a 12 mL vial and added to 100 μL of 1-propanol as internal standard. They were extracted by exposing a 75 μm Carboxen/polydimethylsiloxane (CAR/PDMS) SPME fiber (Supelco, Bellefonte, PA, USA) in the headspace of vials at 40 °C for 50 min and analyzed as reported in Pasqualone et al. [15]. Peak identification was performed by LRI and by computer matching with the reference mass spectra of National Institute of Standards and Technology (NIST) and Wiley libraries. The volatile compounds were quantified by standardizing the peak areas of compounds of interest with the peak area of the internal standard (1-propanol). The analyses were carried out in triplicate.

The polar compounds were recovered by silica gel column chromatography and analyzed by high-performance size-exclusion chromatography (HPSEC) in the conditions reported in Caponio et al. [16]. The content of polar compounds was expressed as g/100 g of oil.

The preparation of the aqueous tomato extract was initially carried out for the tomato analyses: 5 g of product were combined with 50 mL of distilled water, centrifuged at 5000 rpm at room temperature, filtered through filter paper (0.45 μm) and made up to 50 mL volume with distilled water. Then, we proceeded to determine the titratable acidity, pH according to the official methods, of the total soluble solids according to the refractometric method with a PR-201° ATAGO refractometer and color analysis with a CM-700d colorimeter, Konica Minolta, Osaka, (Japan) which uses the CieLab color space. The total color difference ΔE, hue angle, and chroma were evaluated according to Thompson [17].

### 2.3. Pizza Topping Mix and Pizza Sample Analyses

#### 2.3.1. Pizza Topping Mix Qualitative Analyses

The pizza topping mixed samples (tomato sauce + oil A/oil B/oil C) before and after the oven cooking were analyzed for their principal qualitative characteristics. Polyphenol content and total polar compounds were quantified as reported in Section 2.2. The lipid fraction was extracted by the method of Folch et al. [18] and analyzed for oxidation parameters: peroxide number [12], *p*-anisidine value [19], TOTOX [20], and PV/% CDA (conjugated dienoic acid), according to the method reported by Kiritsakis [21]. The Oxitest method is recognized by the AOCS (American Oil Chemists’ Society) and widely used to provide results on the oxidative stability of foods of various origins, especially fats’ and oils’ vegetable origin. The Oxitest reactor subjects the sample to a high-stress environment oxidative to evaluate, in a short period of time, the resistance to oxidation of fats and oils and evaluate the induction period. The induction period is the time it takes to reach the starting point of oxidation, corresponding to a detectable level of rancidity or a sudden change in the oxidation rate. The longer the induction period, the longer will be the oxidative stability over time.

#### 2.3.2. Determination of Volatile Compounds of Pizza Samples

The volatile compounds of pizza samples were determined with the method reported in Section 2.2.

#### 2.3.3. Lipid Extraction

The lipid fraction of pizza was extracted with diethyl ether using a Soxhlet apparatus (SER 148 extraction system, Velp Scientifica Srl, Usmate, Italy).

#### 2.3.4. Determination of Polar Compounds

The polar compounds were determined with the method reported in Section 2.2.

The content of polar compounds was expressed as g/100 g of lipid fraction extracted from pizza. The analyses were carried out in triplicate.

#### 2.3.5. Sensory Analysis of Pizza Samples

Pizza samples (indicated with letters A, B and C in accordance with oil types used) were evaluated by descriptive sensory analysis by a trained panel of 12 judges (3 males and 9 females aged between 19 and 42 years, regular pizza consumers, recruited among departmental students and faculty staff). Samples belonging to each of the three different pizza types were served immediately after cooking in random order unknown to the judges at room temperature, with data averaged over three replicates. Training for pizza sensory attributes was given with one previous tasting session at the same local pizzeria one day before the actual test. Judges rated samples on a 10-point structured scale from 0 to 10 for appearance, aroma, taste, and texture (divided for whole pizza product and topping) descriptors (Table 1), with a 0 indicating the absence of the attribute and 9 an extremely high attribute value. Panelists cleansed their mouths with mineral sparkling water between samples. Mean results are reported in table form and spider plot graphs.

### 2.4. Statistical Analysis

Data are expressed as means ± standard deviation (n = 3). SPSS Software (version 15.0, SPSS Inc., Chicago, IL, USA) was used for data processing. One-way analysis of variance (ANOVA) followed by Tukey’s test for multiple comparisons were applied to the data to determine the presence of significant differences in the chemical and sensory parameters of samples (significance *p* < 0.05).

## 3. Results

### 3.1. Topping Ingredient Analyses

Table 2 shows the results relating to the qualitative characteristics of the oils used for the pizza topping. The detected free acidity was significantly (*p* < 0.01) different, with the lowest values in oil A (0.17 ± 0.05%), followed by oil B (0.30 ± 0.03%) and oil C (0.49 ± 0.01%). These results showed for sample A, as expected, the lowest acidity, due to the deacidification and deodorization processes of the rectified oils that constitute it. The other two samples of oils, B and C, despite having higher percentages of oleic acid, fell well below the maximum limit for extra virgin olive oils provided for by [12], with better results for sample B. This could be explained by the fact that sample B, being a commercial oil, is obtained from a mixture of extra virgin olive oils with different acidity, while oil C, being monovarietal, is strictly influenced by the characteristics of the exclusive cultivar of origin. From this, the free acidity cannot be the only parameter used to establish the quality of an oil, because it is an index that can be easily manipulated by grinding or mixing processes. To evaluate the level of lipid oxidation following cooking of the various ingredients, the peroxides, *p*-anisidine values, spectrophotometric analysis, and induction time were determined. The main products of lipid peroxidation are hydroperoxides, generally referred to as peroxides; therefore, the results the PV parameter give a clear indication of lipid autoxidation [22]. As for the free acidity, sample A showed the lowest results (5.94 ± 0.04 mEq O_2_/kg), due to the submitted treatments, and the other samples (B and C) fell within the limit of 20 mEq O_2_/kg fixed by the European Commission for extra virgin olive oil category.

For further confirmation and deepening of these results, other oxidation parameters were evaluated, such as conjugated dienes and conjugated trienes. Although dienoic acids are less in quantity than monoenoic acids in olive oils, early oxidation occurs mainly in dienoic acids. Consequently, it was interesting to examine the relationship between the value of peroxides (PV) and conjugated dienes (% CDA): the ratio between PV and% CDA normally tends to increase in a system in which oxidation is essentially the result of the oxidation of singlet oxygen with the formation of hydroperoxides in conjugated and unconjugated form [23]. The results on olive oils used in the topping confirmed the differences discussed above denoting the peculiar characteristics of the three samples. These results were reflected in the PV/%CDA of oils, respectively, of 53.05 ± 1.09, 193.62 ± 6.89 and 142.08 ± 14.15. High values of peroxides (primary oxidation) are always an index of low-quality oils, while low values of peroxides do not always indicate good quality [24]: for this reason, the analyses were deepened with the evaluation of *p*-anisidine and the value of TOTOX. The *p*-anisidine value (*p*-AV) is a more reliable and meaningful test, because it measures the secondary oxidation products, which are more stable during the heating process [25]. For the first one, parameter of the secondary oxidation, the obtained results in the three samples were found to be consistent with what was found in the CDA% results and, specifically, by the PV/CDA% ratio in the oils used for the garnish of the pizzas. Relative to the determination of *p*-anisidine, oil C showed the highest value (10.53 ± 0.00), differentiating itself from oil A and B (respectively, 9.10 ± 0.06 and 9.67 ± 0.07). The results of the TOTOX in the oils revealed the highest results in oil B (45.38 ± 1.55), followed by oil C (33.11 ± 0.26) and oil A (20.98 ± 0.14).

The investigation on the total oxidation index revealed the higher quality of the monovarietal extra virgin olive oil then the commercial extra virgin one: this can be linked to the detected qualitative differences in terms of bioactive compounds, such as phenols. Among the extra virgin olive oils used in the present study, the lower polyphenol values of oil B compared to C may be related to the different species of olives used for the production of oils, whereas oil C is monovarietal, with peculiar and exclusive chemical and sensory characteristics. The ANOVA showed significant differences among the three oils: oil C was characterized by the highest concentration of hydroxytyrosol, tyrosol, pinoresinol and apigenin, and in general total phenol content. Having been subjected to thermal rectification processes that led to an almost total degradation of the polyphenolic compounds of the oil, oil A showed the lowest values, followed by oil B (around 110 mg/kg) and oil C (180 mg/kg). The literature reports different levels of polyphenol content in Ottobratica olive oils: our results are similar to those reported by Sicari et al. [26]. Only the concentration of *p*-coumaric acid did not vary among the three oil samples (Table 3).

The volatile compounds showed significant quantitative differences among oil types (Table 4). The olive oil sample, in particular, showed the lowest quantity of volatiles, whereas the extra virgin olive oil Ottobratica cv. showed tenfold the quantity of volatiles of the olive oil. This result was expected, because olive oil is a mix of virgin olive oil (likely in little amounts, hence contributing with little amounts of volatiles), and refined oil, which is submitted, among other processing steps, to a deodorization phase to remove flavors, which unavoidably affects the other volatiles also. The ordinary (multicultivar blend) commercial EVOO had an intermediate content of volatile compounds. A varietal effect on the quali-quantitative profile of volatiles of extra virgin olive oils has been reported in several studies [27,28,29].

The most abundant compound among all the detected ones was (E)-2-hexenal, particularly concentrated in the EVOO from single cultivar Ottobratica Calabrese. This compound has a positive association with olive ripeness and, together with (Z)-3-hexen-1-ol (also abundant in the single cultivar Ottobratica cv. EVOO), is related to a green, grassy note [30].

1-Hexanol, characterized by a green, herbal flavor, was another compound present in noticeable amounts in the Ottobratica cv. extra virgin oil sample. Together with hexanal (which, however, was present in low and similar amounts in the three oils), it originates via the lipoxygenase degradation pathway of linolenic acid [31]. Another lipoxygenase derived volatile was 2,4-hexadienal, having a fatty, sweet, green odor. Nonanal, a typical oxidation marker with a waxy odor and associated with sensory defects, was almost absent in all the oil samples [32,33]. Its absence in the two EVOO oils was due to their good quality, while the absence in olive oil was probably imputable to the deodorization carried out during refining.

Another compound present in relevant amounts in the Ottobratica cv. EVOO was 6-methyl-5-hepten-2-one. Together with benzaldehyde, this compound has been reported in unfiltered EVOO, where it tends to increase with storage, and has been related to the activity of exogenous microorganisms and eventually associated to sensory defects such as musty [34,35]. The presence of a rich residual microflora has been reported in freshly extracted EVOO, being mainly formed by yeasts entrapped in the solid particles and the microdroplets of vegetation water dispersed in the oil phase [36]. While filtration at the end of the extraction process largely removes this suspended material, when the olive oil is not subjected to filtration, the residual microflora may remain partly active in the oil or in the sediment that gradually forms at the bottom of the container, and may significantly contribute to chemical and sensory changes of the oil throughout storage [36]. The Ottobratica cv. EVOO oil, indeed, was not immediately filtered, hence the contact with olive cell residues transferred this volatile compound to the oil. Hexyl acetate was also present, as a derivative of 3-hexen-1-ol.

Table 5 shows the main qualitative analyses carried out on the sample of S. Marzano tomatoes used for the garnish of the Neapolitan pizza. The total acidity was 0.43 ± 0.02% citric acid, total soluble solid content 7.75 ± 0.21° Bx and pH 4.20. The total polyphenol content was quantified in 48.62 ± 0.10 mg/kg of gallic acid with chlorogenic (12.01 ± 0.06 mg/kg), protocatechuic (10.89 ± 0.04 mg/kg) and ferulic (2.74 ± 0.08 mg/kg) acids and rutin (9.09 ± 0.08 mg/kg) as the main phenolic compounds identified on UPLC analysis. The results obtained from the various physicochemical analyses carried out on the tomato showed a good quality and level of antioxidant compounds. In fact, the detected total acidity was completely average for a quality tomato. The total soluble solid content showed a value higher than the minimum required (5° Bx) by the canning industries, and the pH was an index of safety. Flavor is generally related to the relative concentrations of sugars and acids in fruit, especially fructose and citric acid. The best and tastiest combination is a high sugar content and a high acid content. A normal pH range in tomatoes is between 4.0 and 4.5, and the lower the pH, the sweeter or bitterer the fruit will be. A good concentration of acids and sugars correlates with an optimal pH, and can suggest good organoleptic properties of the product as well as analytical ones. The chroma analysis indicates the fullness of color and gave an average result of 9, with positive values also for parameter a* (color variation from green to red), probably given by the presence of carotenoids. The content of chlorogenic acid showed a good level of maturation and processing following a harvest that took place over adequate time [37].

### 3.2. Pizza Topping Mix Analyses

**Figure 1:**
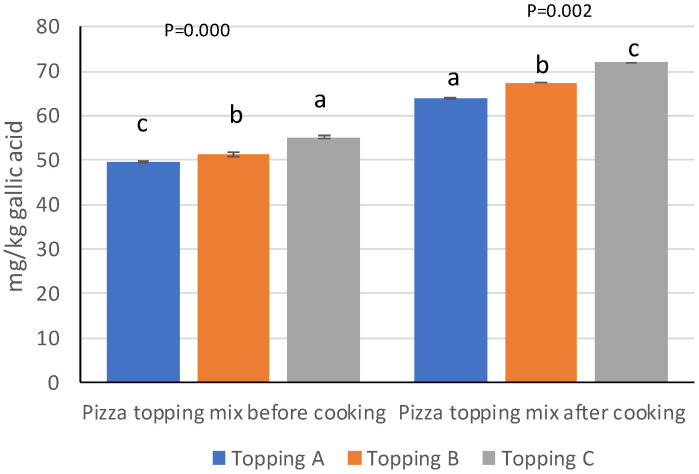
Total polyphenol content of pizza topping mix (tomato sauce + oil A/oil B/oil C) before and after cooking. A (olive oil), B (commercial extra virgin olive oil), C (monovarietal extra virgin olive oil from Ottobratica cv.). Different letters indicate significant differences at *p* < 0.05.

Figure 1 shows the results of the total content of polyphenols in pizza topping mix (tomato + the three different oils) before and after cooking. The three toppings reflected the previously mentioned analytical results for polyphenol content: the topping with oil C in fact was the richest on bioactive compounds (55.24 ± 0.04 and 72.01 ± 0.90 respectively). All the samples showed significant differences from one another.

Regarding the qualitative analysis, the most present compound in the fresh and cooked topping was the protocatechuic acid which was around the value of 9.90 mg/kg for the three toppings before cooking, while it was found in higher concentrations (10.51, 10.79 and 10.51 mg/kg) for the three toppings after the cooking of the pizza, with a slightly higher value in the topping with oil B (Table 6). An increase in post-cooking of rutin is also observed in both topping A and C. In general, there are no substantial differences between the different toppings, but some minimal differences following cooking.

The qualitative and quantitative determination of phenolics in pizza topping mix before and after cooking showed a great influence of the phenolic compounds from tomato with respect to those of the oil: this is mainly due to the interaction between the various compounds of the tomato and the oil promoted precisely by the cooking process. Pernice et al. [38] reported that the heating of a mixture of tomato and olive oil (5%) determined a protective effect by the oil on the antioxidants of the tomato, precisely due to the interaction between the bioactive compounds of the one and the other ingredient. Cooking also increases the disposal of polyphenols [39], which were detected at higher content, and so increased the functional properties of the three pizzas tested. Significant differences were observed in particular for the content of rutin, which is included in dietary adjuvants such as vitamin *p* and is indicated for various health-promoting effects [40].

**Figure 2:**
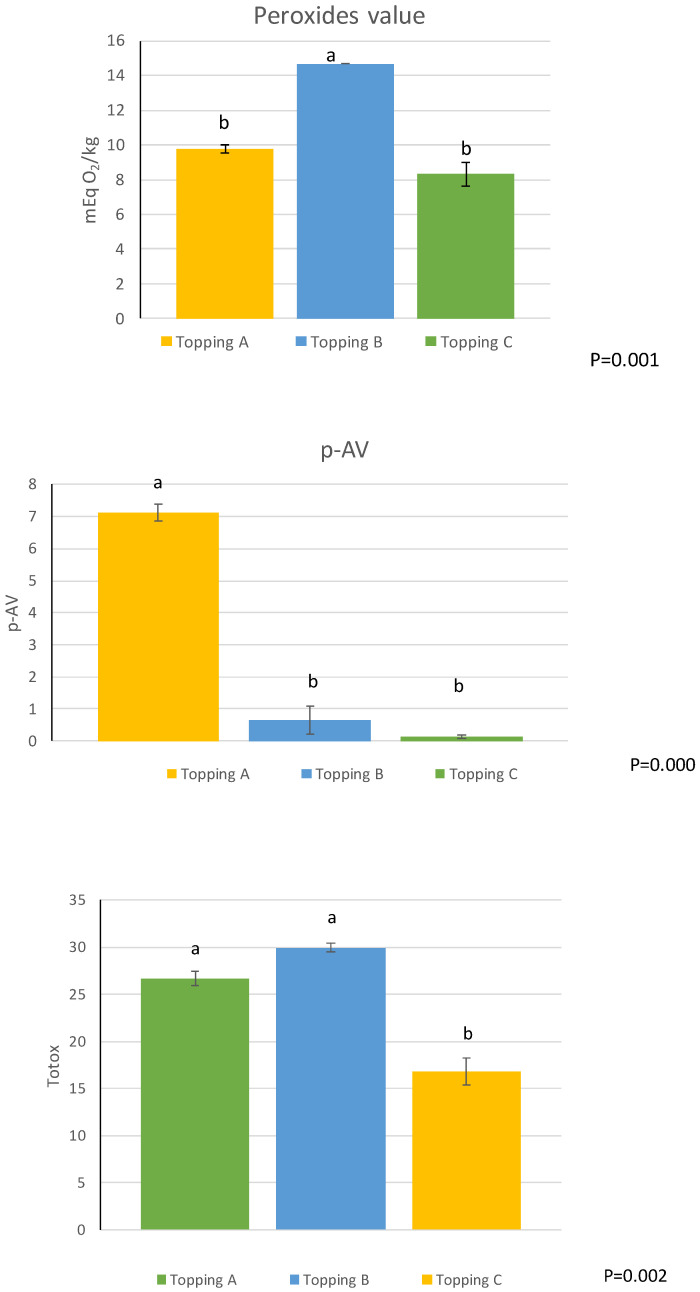
Oxidation indices of topping mix samples after cooking. A (olive oil), B (commercial extra virgin olive oil), C (monovarietal extra virgin olive oil from Ottobratica cv.). Different letters indicate significant differences at *p* < 0.05.

With regard to the analysis of the lipid fraction extracted from the pizza topping after cooking, the resulting peroxide value indicated the topping with oil C of better quality than those composed by oil B. From the *p*-anisidine value, these results were regarding also the topping A after cooking (Figure 2).

**Figure 3:**
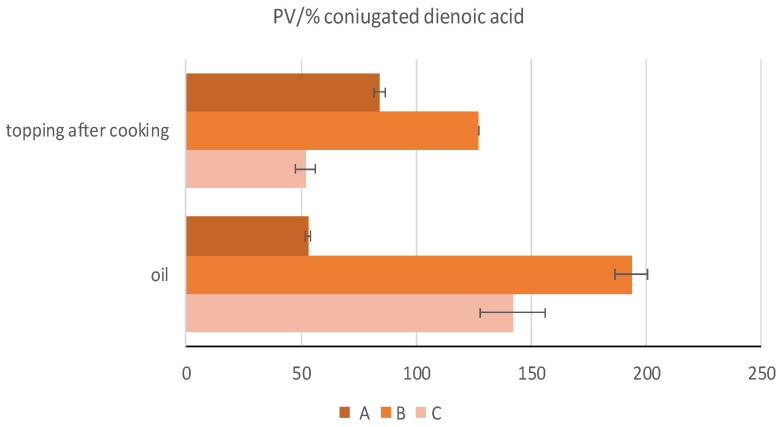
PV/CDA% ratio of fresh olive oil and topping mix samples after cooking. A (olive oil), B (commercial extra virgin olive oil), C (monovarietal extra virgin olive oil from Ottobratica cv.).

The results relating to the lower oxidation state of topping C after cooking led to the consideration that this is probably due to the greater presence and better quality of bioactive compounds (polyphenols, carotenoids and chlorophylls) which, as is known, help prevent and slow down such phenomena. Oxidants, including secondary ones, are reported in Figure 2 for *p*-anisidine values. On the other hand, considering the lipid fraction extracted from the cooked topping, the *p*-anisidine value, as well as the primary oxidation (PV/% CDA) is much lower in the fraction composed of oil C, while it is higher in A and B. This leads us to understand how, despite higher initial values as regards *p*-anisidine, peroxides, % CDA and the PV/CDA% ratio in extra virgin olive oils B and C, after cooking, topping C (composed of tomato and oil extra virgin olive oil C with good levels of bioactive compounds to protect the oxidative advancement of lipids) was found to be qualitatively better than the other two oils (Figure 3). Therefore, by analyzing the TOTOX value, which considers primary and secondary oxidation (peroxides and *p*-anisidine), it is clear that the results of the analysis carried out on crude oil are in line with what was previously observed, i.e., the best result in sample C in terms of lipid oxidation level.

### 3.3. Volatile Compounds of Pizza Samples

Table 7 reports the volatile compounds of pizza seasoned with tomato sauce and the three different types of oil considered in the typical marinara-style pizza. The volatile profile of pizza was much richer compared to the initial oils due to the presence of additional ingredients (flour, yeast and tomato sauce), and due to fermentation activities, thermal reactions and lipid oxidation occurring during pizza processing. However, the three pizza types still showed some significant differences as a function of the type of oil, especially in the levels of 1-hexanol, (Z)-3-hexen-1-ol and (E)-2-hexenal, which decreased due to oxidation (in favor of the formation of other aldehydes and hexanoic acid) [41] but whose levels maintained the same trend observed in the starting oils. In addition, hexanal, nonanal and hexanoic acid, deriving from lipid oxidation, increased compared to the starting oil but were less concentrated in the Ottobratica cv. EVOO, which contained more phenolic compounds with known antioxidant activity.

As for the compounds originating via fermentative activities, ethanol, 2-methyl-1-propanol and 3-methyl-1-butanol are typical yeast products, produced during dough leavening [42]. Relevant amounts of 5-hepten-2-one-6-methyl were detected in all pizza samples, irrespective of the type of oil added. This compound, already present in the starting oil, is present also in tomato products, where derives from the degradation of lycopene; therefore, its increase compared to the starting oil could be due to the tomato sauce used to season the pizza samples [43]. 2-Butanone3-hydroxy, instead, absent in the starting oil, was another fermentation-originated compound [44]. Neither 5-hepten-2-one-6-methyl nor 2-butanone3-hydroxy showed a significant difference among the three pizza types.

As for the compounds related to thermal reactions, due to the typically high temperature of baking adopted to prepare pizza (approximatively 300 °C), this class of compounds comprised 2-methylbutanal, 3-methylbutanal, 2-furancarboxaldehyde (furfural), 5-methyl-2-furancarboxaldehyde (5-methylfurfural), methylpyrazine, ethylpyrazine, 2-pentylfuran, 2-furanmethanol, 2-furanylethanone. All these compounds, absent or present at very low amounts in the three types of oils considered, derived from the Maillard reaction and are commonly reported in the volatile profile of bakery products, being associated with caramel, bread-like flavor [45,46,47,48].

### 3.4. Polar Compounds of the Oil and Pizza

Table 8 shows the content of polar compounds of the oils and of the corresponding pizza samples. These compounds arise from the oxidation and hydrolysis of lipids and their analysis is an effective means to evaluate the quality of any lipid [49,50]. The oxidation products, in particular—namely, triacylglycerol oligopolymers (TAGP) and oxidized triacylglycerols (Ox-TAG)—are implicated with the alteration of the nutritional properties of foods and may cause adverse physiological effects [51]. The diacylglycerols (DAG), instead, originate from lipid hydrolysis.

The oils showed different levels of polar compounds, with the highest values of TAGP and DAG in olive oil, confirming its lower-quality categorization. Between the two EVOOs, the single cultivar Ottobratica cv. oil was of higher quality. The multicultivar EVOO showed the highest content of Ox-TAG.

The results of pizza samples show that a relevant increase of each class of polar compounds occurred during processing, but the difference among oils was maintained, e.g., the less degraded lipid fraction was observed in pizza seasoned with Ottobratica cv. olive oil, which contained higher levels of phenolic antioxidants. Indeed, the preparation of bakery products has been reported to induce the oxidative degradation of lipids [52,53,54].

The observed results were similar to those reported in focaccia, an Italian bakery product similar to pizza [55].

### 3.5. Sensory Analysis

**Figure 4:**
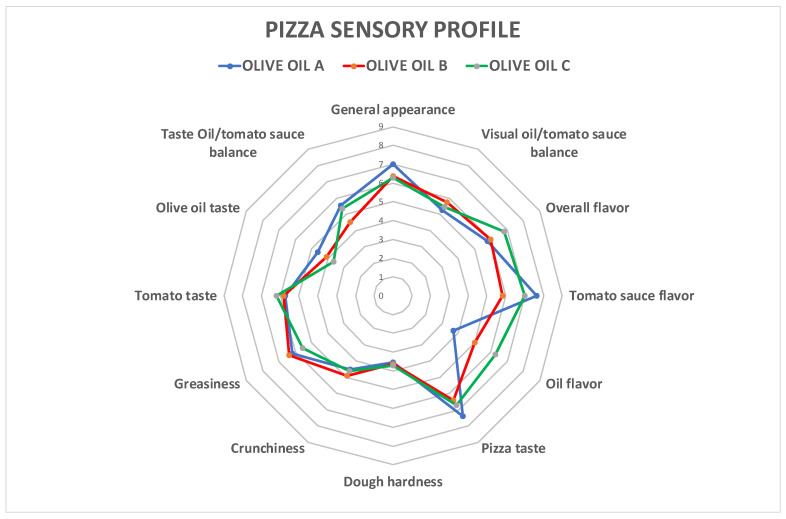
Sensory profile of pizza samples garnished with A (olive oil), B (commercial extra virgin olive oil) and C (monovarietal extra virgin olive oil from Ottobratica cv.).

Sensory profiles for pizza samples produced with the three different oils are shown in Figure 4 and Table 9.

Main descriptors were found to be overall flavor, tomato sauce flavor, and typical pizza taste. The results of the descriptive sensory analysis performed do not show significant differences among samples, since significant differences were found only for the “tomato sauce flavor” and “oil flavor” descriptors. This outcome indicates a possible influence of the different types of olive oil on the product exclusively for the aroma aspect, without influencing other organoleptic aspects of the product. The higher score for oil flavor obtained by pizza with topping C confirmed the previous results on lipid fraction degradation and higher polyphenol content.

## 4. Conclusions

The choice to use different oils to garnish Neapolitan pizza topping is due to the will to understand and evaluate how the characteristics of these can influence the final quality of the topping from the chemical and sensorial points of view. The use of quality ingredients such as San Marzano PDO tomato and a monovarietal olive oil (Ottobratica cv.) with peculiar chemical characteristic content has therefore made it possible to produce a pizza according to the Neapolitan pizza TSG specification with improved health properties that has undergone fewer alterations in lipid fraction over the cooking time, thus maintaining high nutritional value and valuable organoleptic characteristics.

## Figures and Tables

**Table 1:** Pizza sensory descriptor list.

Category	Descriptor	Definition
Appearance	General appearance	Overall appearance of pizza
	Oil–tomato sauce balance	Visual balance between oil and tomato sauce on pizza
Aroma	Overall flavor	Product flavor intensity
	Tomato sauce flavor	Intensity of sauce flavor
	Oil flavor	Intensity of oil flavor
Taste/pizza texture	Pizza taste	Product typical taste intensity
	Dough hardness	Overall hardness of cooked base
	Crunchiness	Teeth cutting resistance intensity
	Greasiness	Mouthfeel greasiness intensity
Taste/topping texture	Tomato taste	Typical tomato taste intensity
	Olive oil taste	Typical olive oil taste intensity
	Oil–tomato sauce balance	Taste balance between olive oil and tomato sauce

**Table 2:** Qualitative characteristics of olive oil used as topping ingredients. A (olive oil), B (commercial extra virgin olive oil), C (monovarietal extra virgin olive oil from Ottobratica cv.).

Samples	A	B	C	Sign.
FA (oleic acid %)	0.17 ± 0.05 ^c^	0.30 ± 0.03 ^b^	0.49 ± 0.00 ^a^	**
PV (mEq O_2_/kg)	5.94 ± 0.04 ^c^	17.85 ± 0.81 ^a^	11.29 ± 0.13 ^b^	**
PV/%CDA	53.05 ± 1.09 ^c^	193.62 ± 6.89 ^a^	142.08 ± 14.15 ^b^	**
*p*-AV	9.10 ± 0.06 ^c^	9.67 ± 0.07 ^b^	10.53 ± 0.00 ^a^	**
TOTOX	20.98 ± 0.14 ^c^	45.38 ± 1.55 ^a^	33.11 ± 0.26 ^b^	**
ΔK	0.06 ± 0.00	0.00 ± 0.00	0.00 ± 0.00	

** Significance at *p* < 0.01. Different letters in the same row indicate significant differences at *p* < 0.05.

**Table 3:** Phenolic composition of olive oils used as topping ingredients. A (olive oil), B (commercial extra virgin olive oil), C (monovarietal extra virgin olive oil from Ottobratica cv.).

Phenolic Compounds (mg/kg)	A	B	C	Sign.
Hydroxytyrosol	2.74 ± 0.02 ^c^	9.13 ± 0.02 ^b^	37.28 ± 0.02 ^a^	**
Tyrosol	1.87 ± 0.05 ^c^	7.67 ± 0.04 ^b^	35.45 ± 0.07 ^a^	**
Omovanillic acid	2.08 ± 0.03 ^a^	1.88 ± 0.02 ^b^	1.88 ± 0.03 ^b^	**
*p*-Coumaric acid	0.23 ± 0.01	0.19 ± 0.01	0.19 ± 0.01	ns
Pinoresinol	1.18 ± 0.01 ^c^	4.09 ± 0.02 ^b^	11.44 ± 0.01 ^a^	**
Oleuropein	0.00 ± 0.00 ^c^	4.08 ± 0.02 ^a^	2.74 ± 0.01 ^b^	**
Apigenin	0.32 ± 0.01 ^c^	1.60 ± 0.03 ^b^	2.18 ± 0.03 ^a^	**
TPC	12.63 ± 1.57 ^c^	109.39 ± 2.38 ^b^	179.01 ± 10.23 ^a^	**

** Significance at *p* < 0.01; ns, not significant. Different letters in the same row indicate significant differences at *p* < 0.05.

**Table 4:** Volatile compounds of the oils used in pizza seasoning. A (olive oil), B (commercial extra virgin olive oil), C (monovarietal extra virgin olive oil from Ottobratica cv.).

Volatile Compound (µg/g)	Type of Oil		
	A	B	C
Alcohols			
1-Hexanol	0.80 ± 0.06 ^c^	1.50 ± 0.01 ^b^	7.26 ± 0.77 ^a^
(Z)-3-Hexen-1-ol	1.75 ± 0.21 ^c^	7.34 ± 0.10 ^b^	24.82 ± 2.36 ^a^
Aldehydes			
2-Methylbutanal	0.20 ± 0.02 ^b^	0.77 ± 0.07 ^a^	n.d.
3-Methylbutanal	1.21 ± 0.12 ^a^	0.92 ± 0.10 ^b^	n.d.
Pentanal	n.d.	4.32 ± 0.50	n.d.
Hexanal	1.52 ± 0.18 ^ab^	1.82 ± 0.14 ^a^	1.43 ± 0.14 ^b^
(E)-2-Hexenal	4.07 ± 0.51 ^c^	11.25 ± 0.64 ^b^	65.55 ± 4.00 ^a^
Octanal	n.d.	0.97 ± 0.13	n.d.
Nonanal	0.05 ± 0.02 ^c^	0.60 ± 0.10 ^a^	0.32 ± 0.02 ^b^
2,4-Hexadienal	0.13 ± 0.02 ^c^	0.67 ± 0.14 ^b^	4.33 ± 0.32 ^a^
Benzaldehyde	0.28 ± 0.01 ^b^	0.50 ± 0.08 ^a^	0.31 ± 0.08 ^ab^
Ketones			
3-Pentanone	n.d.	0.75 ± 0.03 ^b^	1.99 ± 0.12 ^a^
2-Octanone	0.11 ± 0.01	n.d.	n.d.
6-Methyl-5-hepten-2-one	0.43 ± 0.04 ^c^	1.32 ± 0.16 ^b^	4.82 ± 0.47 ^a^
2-Nonanone	0.04 ± 0.01 ^c^	0.44 ± 0.02 ^a^	0.35 ± 0.02 ^b^
Carboxylic acids			
Acetic acid	0.71 ± 0.06 ^a^	0.72 ± 0.05 ^a^	0.04 ± 0.01 ^b^
Esters			
Methyl acetate	1.47 ± 0.14 ^b^	2.64 ± 0.57 ^a^	n.d.
Ethyl acetate	n.d.	1.69 ± 0.04 ^a^	0.45 ± 0.03 ^b^
Hexyl acetate	0.89 ± 0.05 ^c^	2.74 ± 0.05 ^b^	3.67 ± 0.48 ^a^

Different letters in the same row indicate significant differences at *p* < 0.05; n.d., not detected.

**Table 5:** Qualitative characteristics of San Marzano PDO tomato samples used as topping ingredient.

San Marzano PDO Tomato	
Total acidity (% citric acid)	0.43 ± 0.02
pH	4.17 ± 0.01
SST (°Brix)	7.75 ± 0.21
L *	45.46 ± 0.49
a *	6.02 ± 0.71
b *	6.70 ± 0.60
Chroma	9.01 ± 0.15
TPC (mg/kg gallic acid)	48.62 ± 0.10
Protocatechuic acid (mg/kg)	10.89 ± 0.04
Chlorogenic acid (mg/kg)	12.01 ± 0.06
Ferulic acid (mg/kg)	2.74 ± 0.08
Rutin (mg/kg)	9.09 ± 0.08

**Table 6:** Phenolic composition of topping mix samples A (olive oil), B (commercial extra virgin olive oil), C (monovarietal extra virgin olive oil from Ottobratica cv.).

	Topping before Cooking			Sign.	Topping after Cooking			Sign.
	A	B	C		A	B	C	
Protocatechuic acid	9.92 ± 0.02	9.90 ± 0.21	9.58 ± 0.12	ns	10.51 ± 0.09	10.79 ± 0.16	10.51 ± 0.05	ns
Chlorogenic acid	9.57 ± 0.10	9.77 ± 2.72	9.35 ± 0.08	ns	9.71 ± 0.04	9.58 ± 0.03	9.72 ± 0.10	ns
Ferulic acid	2.72 ± 0.03	2.72 ± 0.03	2.76 ± 0.01	ns	2.02 ± 0.03 ^b^	2.44 ± 0.12 ^b^	2.99 ± 0.02 ^a^	**
Rutin	7.42 ± 0.03 ^b^	8.20 ± 0.07 ^a^	7.72 ± 0.01 ^b^	**	8.42 ± 0.09 ^a^	7.53 ± 0.09 ^b^	8.23 ± 0.08 ^a^	**

** Significance at *p* < 0.01; ns, not significant. Different letters in the same row indicate significant differences at *p* < 0.05.

**Table 7:** Volatile compounds of pizza seasoned with tomato sauce and three different types of oil (marinara-style pizza): A (olive oil), B (commercial extra virgin olive oil), C (monovarietal extra virgin olive oil from Ottobratica cv.).

Volatile Compound (µg/g)	Type of Pizza		
	A	B	C
Alcohols			
Ethanol	23.04 ± 1.78 ^a^	22.39 ± 2.19 ^a^	25.10 ± 1.05 ^a^
2-Methyl-1-propanol	6.03 ± 0.13 ^c^	8.02 ± 0.09 ^b^	8.83 ± 0.14 ^a^
3-Methyl-1-butanol	13.66 ± 1.31 ^ab^	10.62 ± 1.06 ^b^	14.93 ± 1.49 ^a^
1-Hexanol	0.98 ± 0.06 ^c^	1.30 ± 0.04 ^b^	3.48 ± 0.38 ^a^
(Z)-3-Hexen-1-ol	0.97 ± 0.05 ^c^	3.88 ± 0.06 ^b^	6.93 ± 0.05 ^a^
Aldehydes			
2-Methylbutanal	10.62 ± 0.32 ^a^	10.42 ± 0.24 ^a^	10.96 ± 0.11 ^a^
3-Methylbutanal	2.14 ± 0.11 ^a^	2.40 ± 0.27 ^a^	1.66 ± 0.20 ^b^
Hexanal	4.52 ± 0.14 ^a^	4.86 ± 0.19 ^a^	2.12 ± 0.09 ^b^
(E)-2-Hexenal	0.09 ± 0.03 ^c^	3.43 ± 0.33 ^b^	10.26 ± 1.51 ^a^
(E,E)-2,4-Hexadienal	n.d.	1.48 ± 0.03 ^a^	0.46 ± 0.08 ^b^
Nonanal	1.91 ± 0.37 ^a^	1.70 ± 0.22 ^a^	0.54 ± 0.04 ^b^
2-Furancarboxaldehyde	11.68 ± 1.21 ^b^	18.35 ± 0.95 ^a^	11.29 ± 1.14 ^b^
Benzaldehyde	3.33 ± 0.36 ^ab^	3.86 ± 0.34 ^a^	3.02 ± 0.26 ^b^
5-methyl-2-furancarboxaldehyde	1.77 ± 0.15 ^b^	3.55 ± 0.35 ^a^	1.39 ± 0.12 ^b^
Ketones			
Acetone	3.28 ± 0.38 ^a^	2.77 ± 0.30 ^ab^	2.46 ± 0.28 ^b^
3-Hydroxy-2-butanone	8.87 ± 0.78 ^a^	7.10 ± 2.62 ^a^	8.34 ± 3.02 ^a^
5-Hepten-2-one-6-methyl	9.42 ± 2.15 ^a^	7.66 ± 3.81 ^a^	8.55 ± 2.67 ^a^
2-Nonanone	0.09 ± 0.01 ^b^	0.22 ± 0.03 ^a^	0.22 ± 0.03 ^a^
Acids			
Acetic acid	15.57 ± 3.30 ^a^	20.60 ± 1.70 ^a^	16.86 ± 1.13 ^a^
Propanoic acid	n.d.	0.77 ± 0.22 ^a^	0.50 ± 0.32 ^ab^
Pentanoic acid	1.30 ± 0.25 ^b^	2.86 ± 0.27 ^a^	1.02 ± 0.09 ^b^
Hexanoic acid	0.54 ± 0.05 ^a^	0.09 ± 0.01 ^b^	0.10 ± 0.01 ^b^
Esters			
Ethyl acetate	2.16 ± 0.25 ^b^	3.27 ± 0.39 ^a^	1.96 ± 0.49 ^b^
3-Hexen-1-ol, acetate	1.31 ± 0.07 ^c^	5.87 ± 0.60 ^a^	3.62 ± 0.08 ^b^
Pyrazines			
Pyrazine	1.71 ± 0.14 ^b^	2.67 ± 0.35 ^a^	0.16 ± 0.14 ^c^
Methylpyrazine	4.93 ± 0.42 ^a^	4.67 ± 0.28 ^a^	1.13 ± 0.09 ^b^
Ethylpyrazine	4.42 ± 1.11 ^a^	2.84 ± 0.15 ^b^	2.11 ± 0.12 ^c^
Furans			
1-(2-furanyl)-Ethanone	0.91 ± 0.18 ^b^	1.36 ± 0.08 ^a^	0.80 ± 0.03 ^b^
2-Pentylfuran	0.23 ± 0.03 ^b^	1.06 ± 0.21 ^a^	0.42 ± 0.07 ^b^
2-Furanmethanol	1.14 ± 0.19 ^b^	2.82 ± 0.15 ^a^	1.37 ± 0.37 ^b^

Different letters in the same row indicate significant differences at *p* < 0.05.

**Table 8:** Polar compounds of different oils used in pizza seasoning, before and after baking. A (olive oil), B (commercial extra virgin olive oil), C (monovarietal extra virgin olive oil from Ottobratica cv.).

Polar Compound (g/100 g)	A	B	C
Uncooked oil			
TAGP	0.16 ± 0.01 ^aB^	0.09 ± 0.01 ^bB^	0.05 ± 0.01 ^bB^
Ox-TAG	0.61 ± 0.01 ^bB^	0.73 ± 0.03 ^aB^	0.49 ± 0.02 ^cB^
DAG	1.96 ± 0.08 ^aB^	1.62 ± 0.05 ^bB^	1.74 ± 0.07 ^abB^
Pizza *			
TAGP	0.29 ± 0.01 ^aA^	0.21 ± 0.01 ^bA^	0.11 ± 0.01 ^cA^
Ox-TAG	1.51 ± 0.01 ^aA^	1.05 ± 0.02 ^bA^	0.89 ± 0.02 ^cA^
DAG	4.39 ± 0.09 ^aA^	3.02 ± 0.01 ^bA^	2.36 ± 0.03 ^cA^

* Seasoned with oil and tomato sauce, without mozzarella cheese. TAGP = triacylglycerol oligopolymers; Ox-TAG = oxidized triacylglycerols; DAG = diacylglycerols. Different lowercase letters in row indicate significant differences among oils or pizza types (*p* < 0.05); different uppercase letters in columns indicate significant differences for the same compound in uncooked oil and in the corresponding pizza.

**Table 9:** Descriptive sensory analysis results for pizza samples garnished with A (olive oil), B (commercial extra virgin olive oil), C (monovarietal extra virgin olive oil from Ottobratica cv.).

Descriptor	A	B	C	Sig.
Appearance				
General appearance	7.00 ± 1.21 ^a^	6.50 ± 1.83 ^a^	6.33 ± 1.50 ^a^	ns
Oil/tomato sauce balance	5.17 ± 1.64 ^a^	5.58 ± 1.31 ^a^	5.33 ± 1.16 ^a^	ns
Aroma				
Overall flavor	6.00 ± 1.81 ^a^	6.17 ± 1.59 ^a^	6.83 ± 1.12 ^a^	ns
Tomato sauce flavor	7.67 ± 1.16 ^a^	6.00 ± 2.45 ^b^	7.08 ± 1.83 ^ab^	**
Oil flavor	3.58 ± 1.44 ^a^	4.92 ± 1.88 ^ab^	5.92 ± 2.28 ^b^	**
Taste/pizza texture				
Pizza taste	6.92 ± 2.50 ^a^	6.67 ± 1.56 ^a^	6.92 ± 2.15 ^a^	ns
Dough hardness	3.50 ± 1.31 ^a^	3.58 ± 1.68 ^a^	3.58 ± 1.88 ^a^	ns
Crunchiness	4.42 ± 2.11 ^a^	4.75 ± 2.73 ^a^	4.50 ± 2.75 ^a^	ns
Greasiness	6.00 ± 1.81 ^a^	6.25 ± 2.60 ^a^	5.25 ± 2.56 ^a^	ns
Taste/topping texture				
Tomato taste	5.58 ± 2.19 ^a^	5.67 ± 2.15 ^a^	6.00 ± 2.34 ^a^	ns
Olive oil taste	4.33 ± 1.97 ^a^	3.75 ± 2.77 ^a^	3.42 ± 2.50 ^a^	ns
Oil/tomato sauce balance	5.42 ± 1.83 ^a^	4.50 ± 2.02 ^a^	5.25 ± 1.87 ^a^	ns

** Significance at *p* < 0.01; ns, not significant. Different letters in row indicate significant differences at *p* < 0.05.

## Data Availability

Data is contained within the article.
